# Supplementary material for: Pythia: Non-random DNA repair allows predictable CRISPR/Cas9 integration and gene editing
Source: bioRxiv. 2024 Sep 23:2024.09.23.614424. Preprint. [Version 1] doi: 10.1101/2024.09.23.614424 (PMC11463480; doi:10.1101/2024.09.23.614424)
Supplement: Supplement 2 [file NIHPP2024.09.23.614424v1-supplement-2.pdf]

bioRxiv preprint doi: https://doi.org/10.1101/2024.09.23.614424; this version posted September 23, 2024. The copyright holder for this preprint (which was not certified by peer review) is the author/funder, who has granted bioRxiv a license to display the preprint in perpetuity. It is made available under aCC-BY 4.0 International license.

Supplementary information

Supplementary Figures

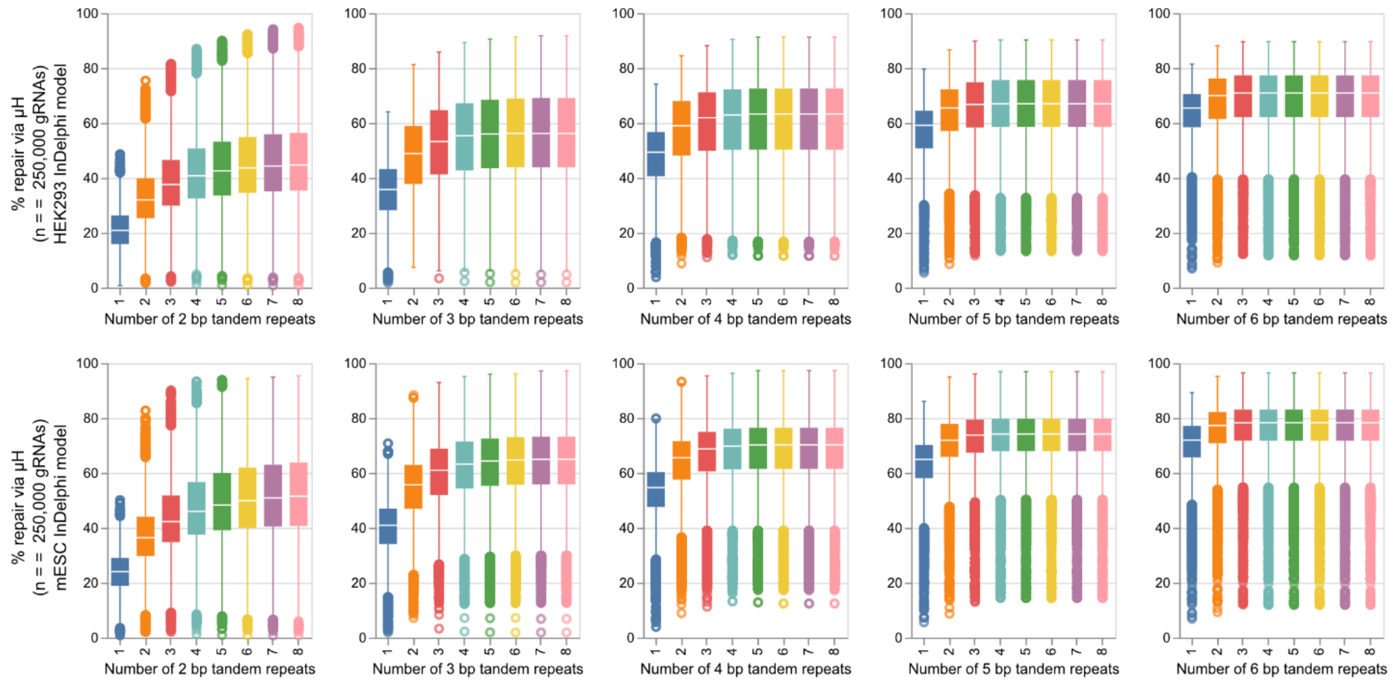

Supplementary Figure 1: Computational modelling of predicted %repair by µH in relationship to a number and length of tandem repeats. For 250,000 gRNAs binding the human genome, we modelled the expected editing outcomes when adding local sequence context left of the CRISPR/Cas9-mediated DSB to the right of the cut. Predicted %repair by µH is defined as any repair that mobilizes any of the available tandem repeats. This was computed using the InDelphi-HEK293T model (Top) and the InDelphi-mESC model (bottom).

bioRxiv preprint doi: https://doi.org/10.1101/2024.09.23.614424; this version posted September 23, 2024. The copyright holder for this preprint (which was not certified by peer review) is the author/funder, who has granted bioRxiv a license to display the preprint in perpetuity. It is made available under aCC-BY 4.0 International license.

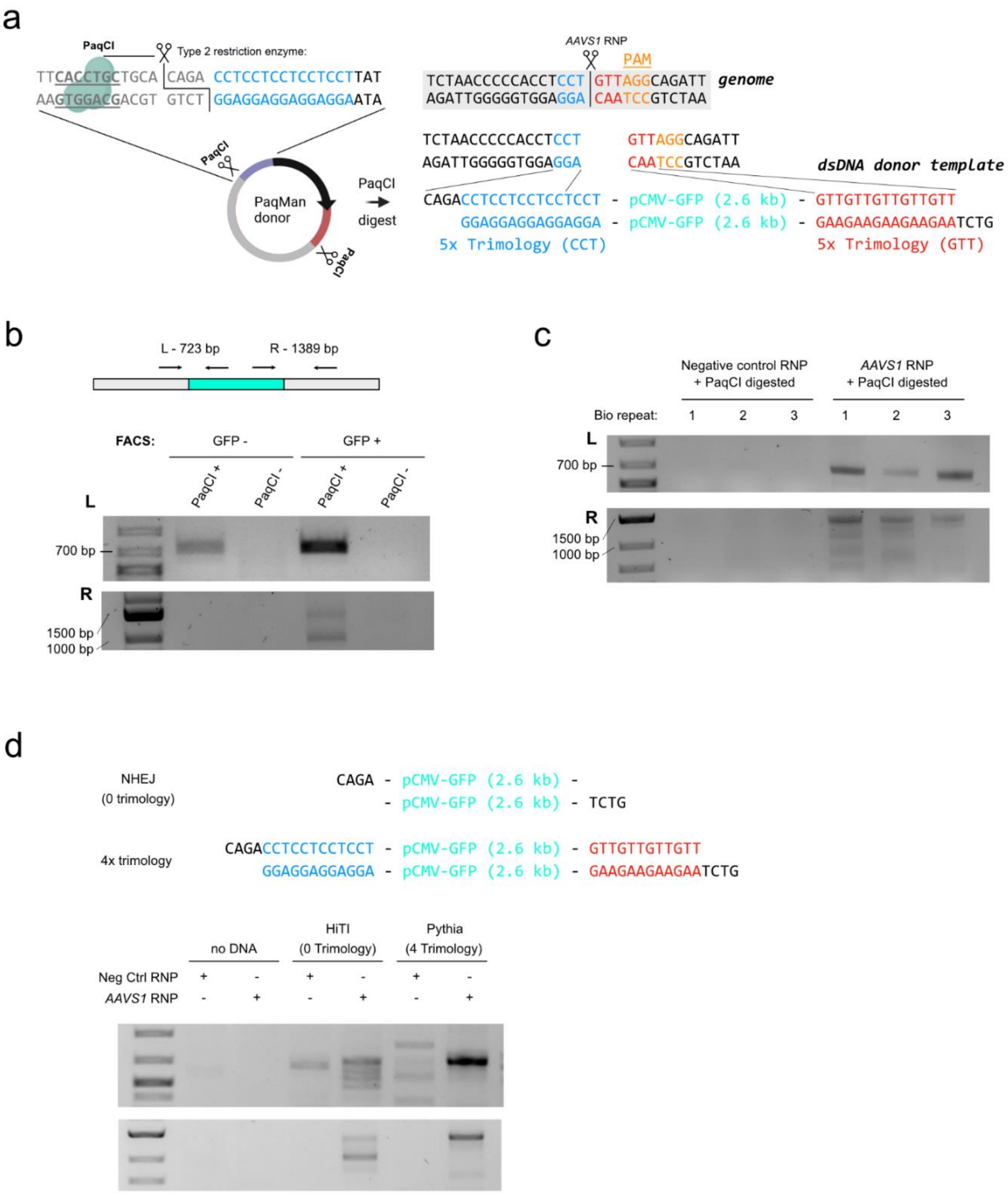

Supplementary Figure 2: Trimology integration in the *AAVS1* locus (a) Scheme of CRISPR/Cas9 integration strategy. The donor plasmid consists out of pCMV-eGFP expression cassette, flanked by 5x Trimology arms and inverted PaqCI restriction enzyme binding sites. dsDNA donor template is liberated by *in vitro* PaqCI digest, where this type II restriction enzyme provides a cut away from its recognition sequence, allowing fully customizable edges of the dsDNA donor template. This digest is co-delivered with *AAVS1* RNP into HEK293T cells. **(b)** Only with digested (PaqCI-linearized), but not with undigested, can we amplify 5' (L) and 3' (R) junction products. This was performed on lysis from cells FACS-sorted into GFP- and GFP+ subpopulations. **(c)** 5' (L) and 3' (R) junction products can only be amplified when co-delivering *AAVS1*, and not when co-delivering negative control RNP. **(d)** dsDNA repair templates as obtained after *in vitro* PaqCI digest of donor plasmids. This repair templates where co-delivered together with either negative control RNP or *AAVS1* RNP and contain either 4x trimology repair arms or 0x trimology repair arms (NHEJ).

bioRxiv preprint doi: https://doi.org/10.1101/2024.09.23.614424; this version posted September 23, 2024. The copyright holder for this preprint (which was not certified by peer review) is the author/funder, who has granted bioRxiv a license to display the preprint in perpetuity. It is made available under aCC-BY 4.0 International license.

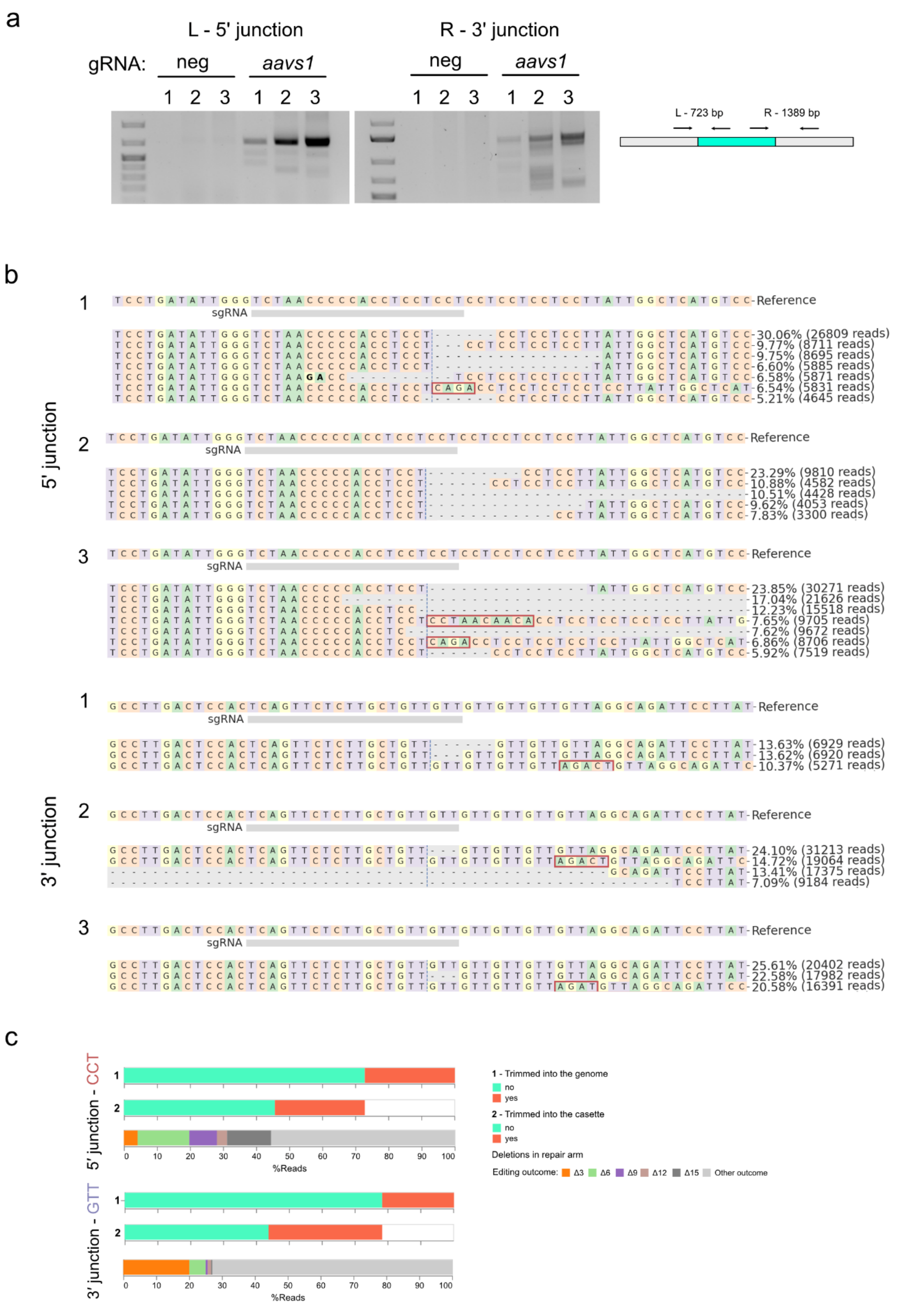

Supplementary Figure 3: Targeted deep amplicon sequencing of genome-transgene boundary products. (a) 5' (L) and 3' (R) junction products can only be amplified when co-delivering *AAVS1*, and not when co-delivering negative control RNP. Numbers 1 through 3 denote biological repeats (*n*=3). **(b)** CRISPResso2 analysis of next-generation sequencing of 5' (L) and 3' (R) junction products. **(c)** Visualisation of genome editing outcomes on both genome-transgene junctions demonstrating trimming both into the genome (1) and the transgene cassette (2). For each read in which no trimming into the genome or the cassette was observed, the number of deletions in the repair arms are shown.

bioRxiv preprint doi: https://doi.org/10.1101/2024.09.23.614424; this version posted September 23, 2024. The copyright holder for this preprint (which was not certified by peer review) is the author/funder, who has granted bioRxiv a license to display the preprint in perpetuity. It is made available under aCC-BY 4.0 International license.

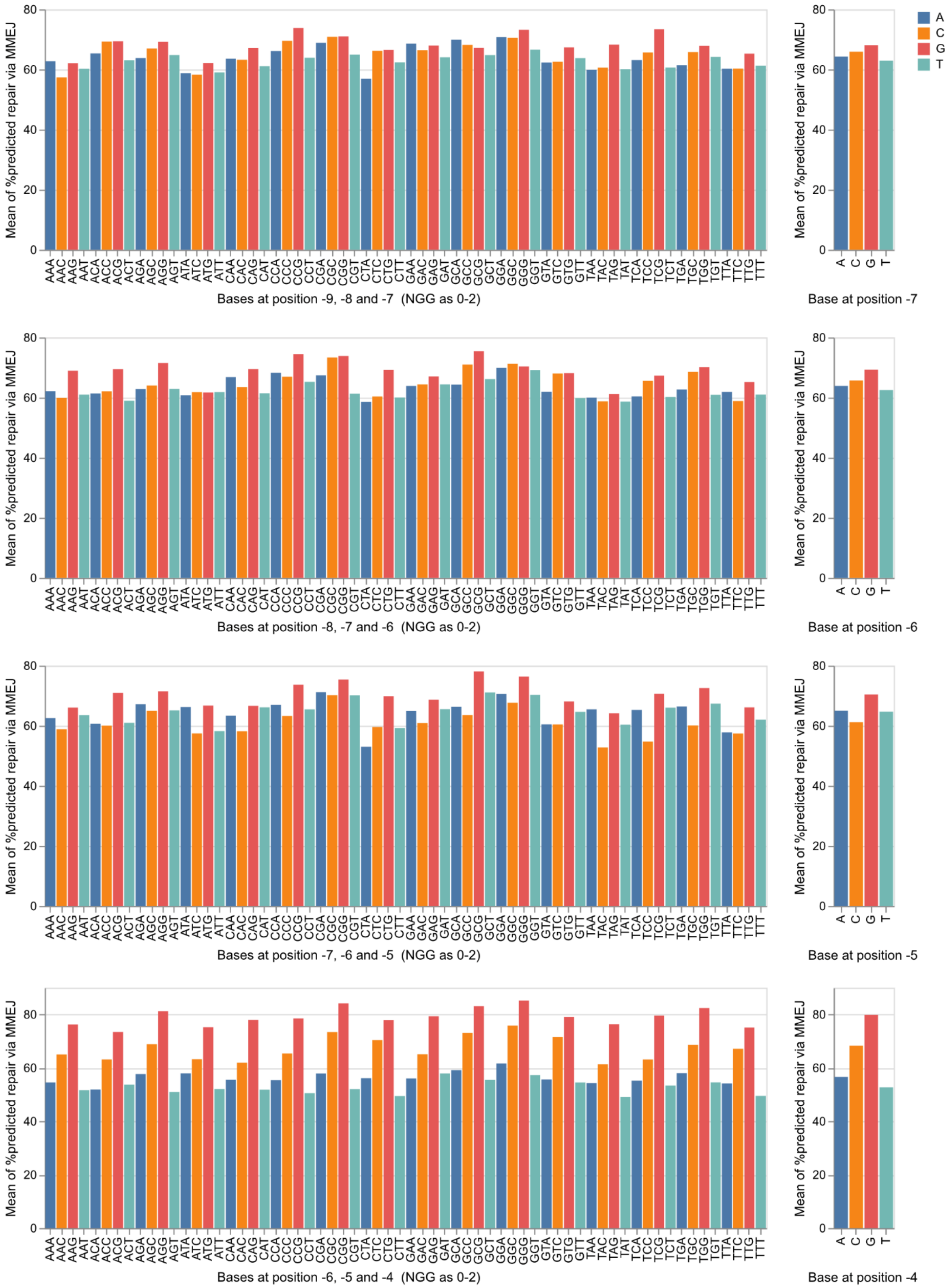

Supplementary Figure 4: Modelling the predicted percentage of repair via MMEJ based on the base-composition of the gRNA binding site in the genome reveals that base composition at position -4 increases the % of gene editing outcomes by MMEJ for gRNAs with PAM "NGG". We performed an exome-wide analysis across the human coding genome using the InDelphi-HEK293 model retrieving 10,813,171 unique gRNAs. A subset of 500,00 gRNAs was randomly selected for plotting. This reveals that there is an enrichment for expected % of gene editing outcomes by MMEJ when the base at position -4 is a C or G, when compared to A or T. Such enrichment is not apparent for the positions -5 through -9 (counting the NGG PAM as nucleotides 0-2).

bioRxiv preprint doi: https://doi.org/10.1101/2024.09.23.614424; this version posted September 23, 2024. The copyright holder for this preprint (which was not certified by peer review) is the author/funder, who has granted bioRxiv a license to display the preprint in perpetuity. It is made available under aCC-BY 4.0 International license.

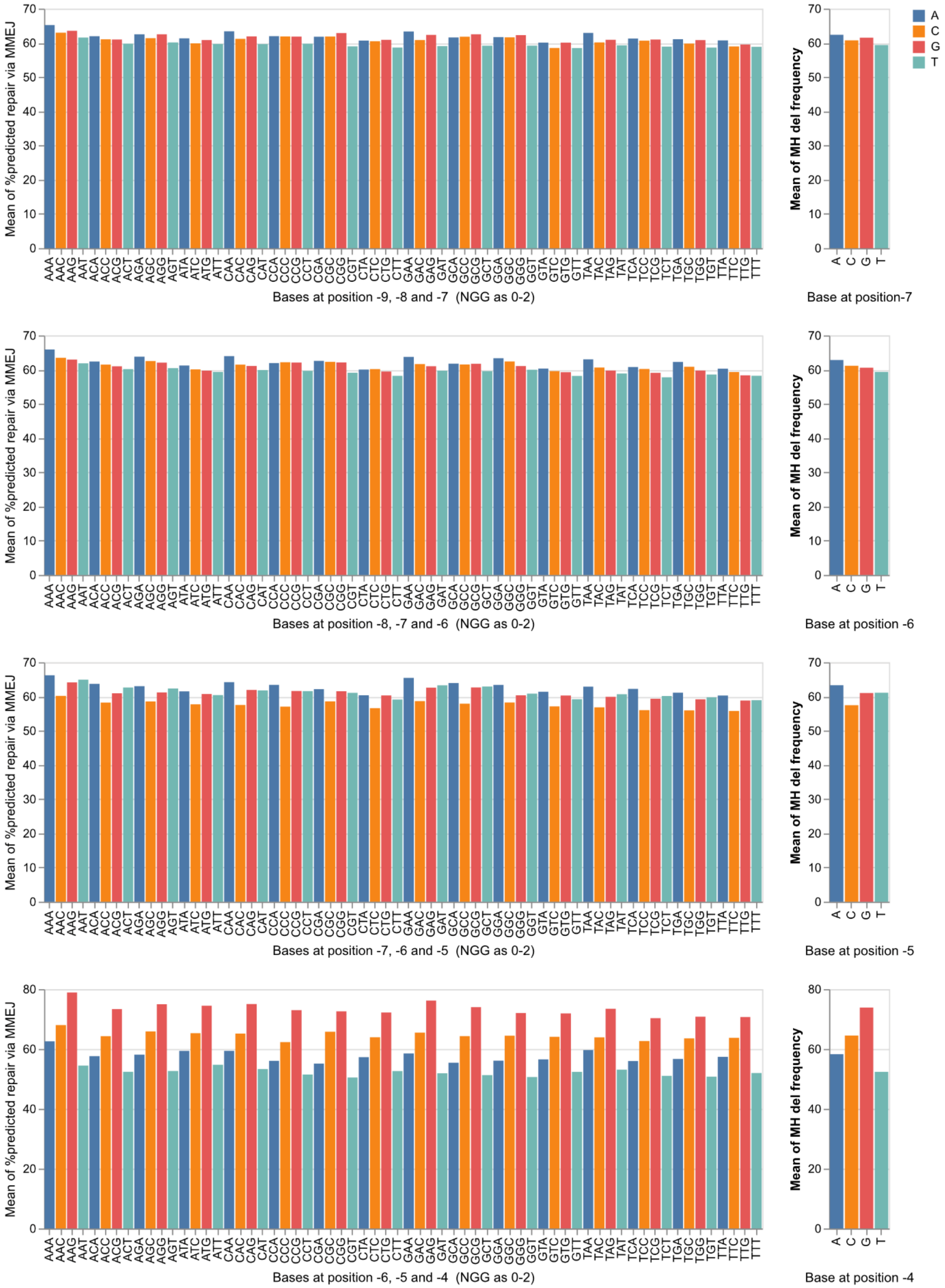

Supplementary Figure 5: Modelling the predicted percentage of repair via MMEJ based on the base-composition of the gRNA binding site in the genome reveals that base composition at position -4 increases the % of gene editing outcomes by MMEJ for gRNAs with PAM "NAA". We performed an analysis across a subset of the human coding exome using the InDelphi-HEK293 model retrieving 1,751,128 unique gRNAs. A subset of 500,00 gRNAs was randomly selected for plotting. This reveals that there is an enrichment for expected % of gene editing outcomes by MMEJ when the base at position -4 is a C or G, when compared to A or T. Such enrichment is not apparent for the positions -5 through -9 (counting the NGG PAM as nucleotides 0-2).

bioRxiv preprint doi: https://doi.org/10.1101/2024.09.23.614424; this version posted September 23, 2024. The copyright holder for this preprint (which was not certified by peer review) is the author/funder, who has granted bioRxiv a license to display the preprint in perpetuity. It is made available under aCC-BY 4.0 International license.

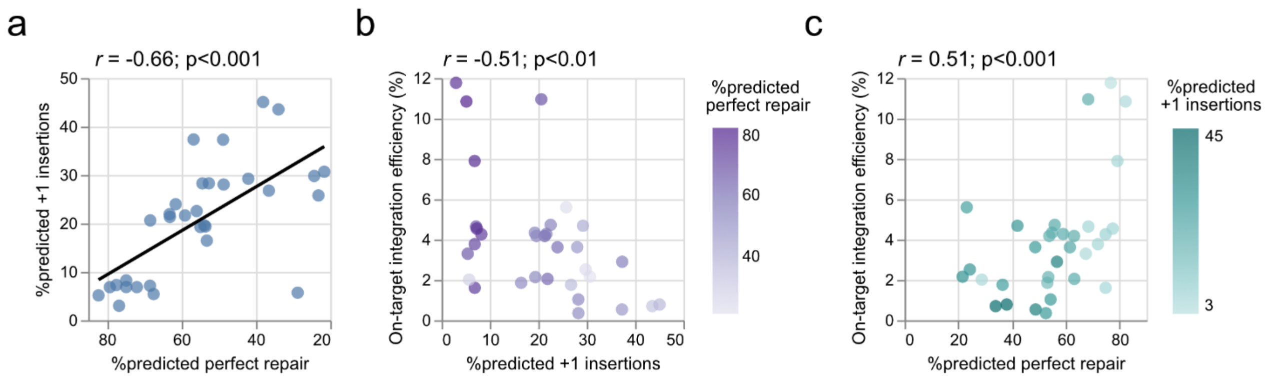

Supplementary figure 6: Correlating experimental on-target integration efficiencies to InDelphi predictions. (a) There is an inverse correlation between the % of predicted perfect repair (defined as any repair mobilizing a trimology repeat) and the % of predicted +1 insertions. **(b-c)** As such, on-target integration efficiencies decrease when there is a higher % of prediction for +1 insertions and increase when there is a higher % of predicted perfect repair.

bioRxiv preprint doi: https://doi.org/10.1101/2024.09.23.614424; this version posted September 23, 2024. The copyright holder for this preprint (which was not certified by peer review) is the author/funder, who has granted bioRxiv a license to display the preprint in perpetuity. It is made available under aCC-BY 4.0 International license.

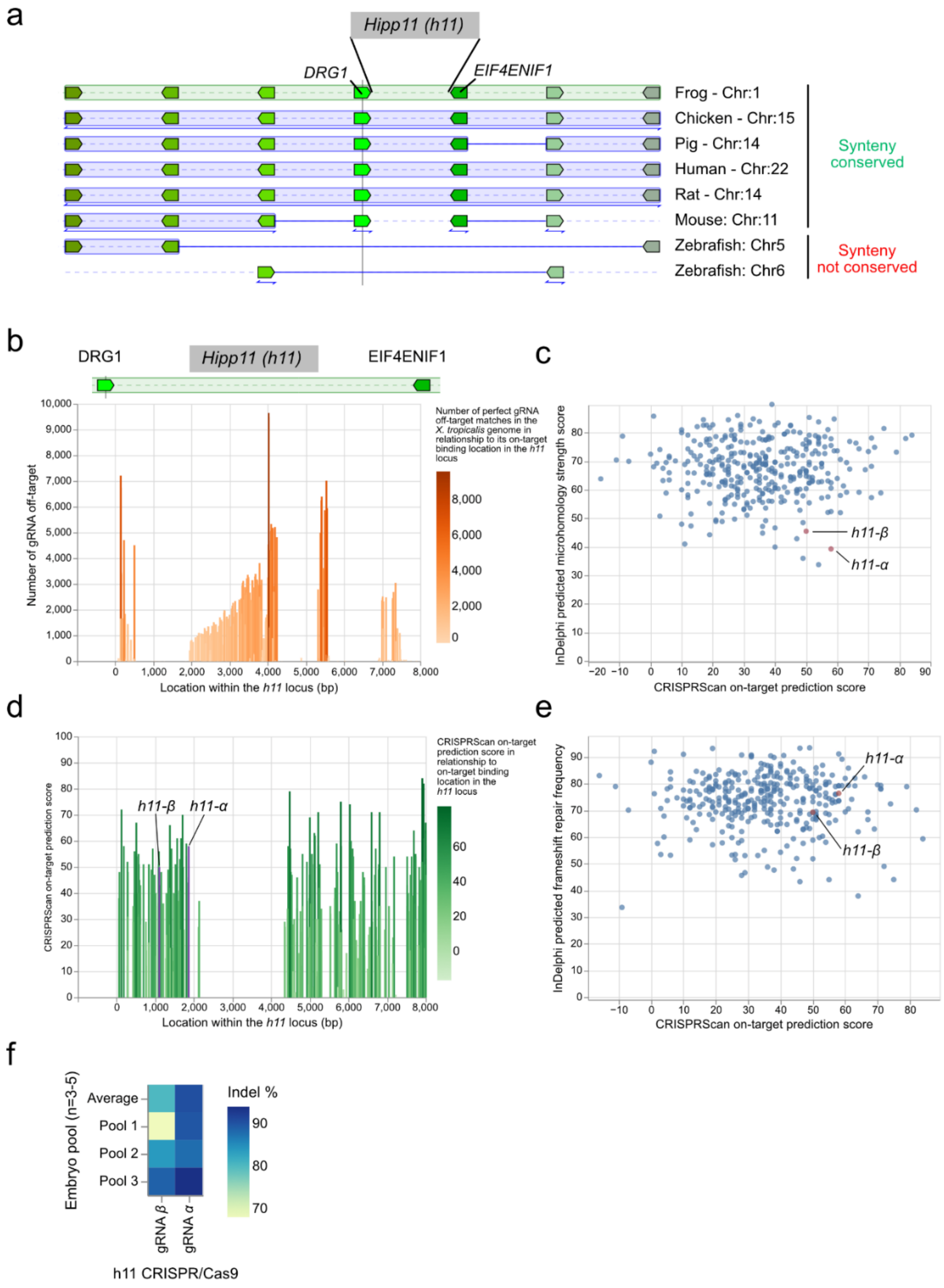

Supplementary figure 7: The *Xenopus tropicalis hipp11* locus can be gene edited by CRISPR/Cas9. (a) Using genome synteny (Genomicus[35]), we identify conservation of the intergenic region between *drg1* and *eif4enif1* corresponding to the *Hipp11* (*H11*) transgene landing site previously described in human, mouse and pig. Of note, synteny here is not conserved in the teleost lineage. **(b)** 55.3% (447 gRNAs/809) of potential gRNAs targeting this region have an unacceptable off-target profile with >1 perfect off-target match in the *Xenopus tropicalis* genome. **(c-e)** For the remaining gRNAs, we calculated via InDelphi-mESC the predicted frequency of editing outcomes via MMEJ, predicted frameshift repair frequency and the on-target CRISPRScan score to identify two suitable gRNA target sites (*h11-α* and *h11-β*) spaced 767bp apart. **(f)** One-cell stage *X. tropicalis* embryos were injected with CRISPR/Cas9 targeting either *h11-α* or *h11-β*. Embryos were grown until stage 23, lysed (pools of 3 to 5 embryos) and genome editing efficiencies are shown, as determined by sanger sequencing and trace deconvolution.

bioRxiv preprint doi: https://doi.org/10.1101/2024.09.23.614424; this version posted September 23, 2024. The copyright holder for this preprint (which was not certified by peer review) is the author/funder, who has granted bioRxiv a license to display the preprint in perpetuity. It is made available under aCC-BY 4.0 International license.

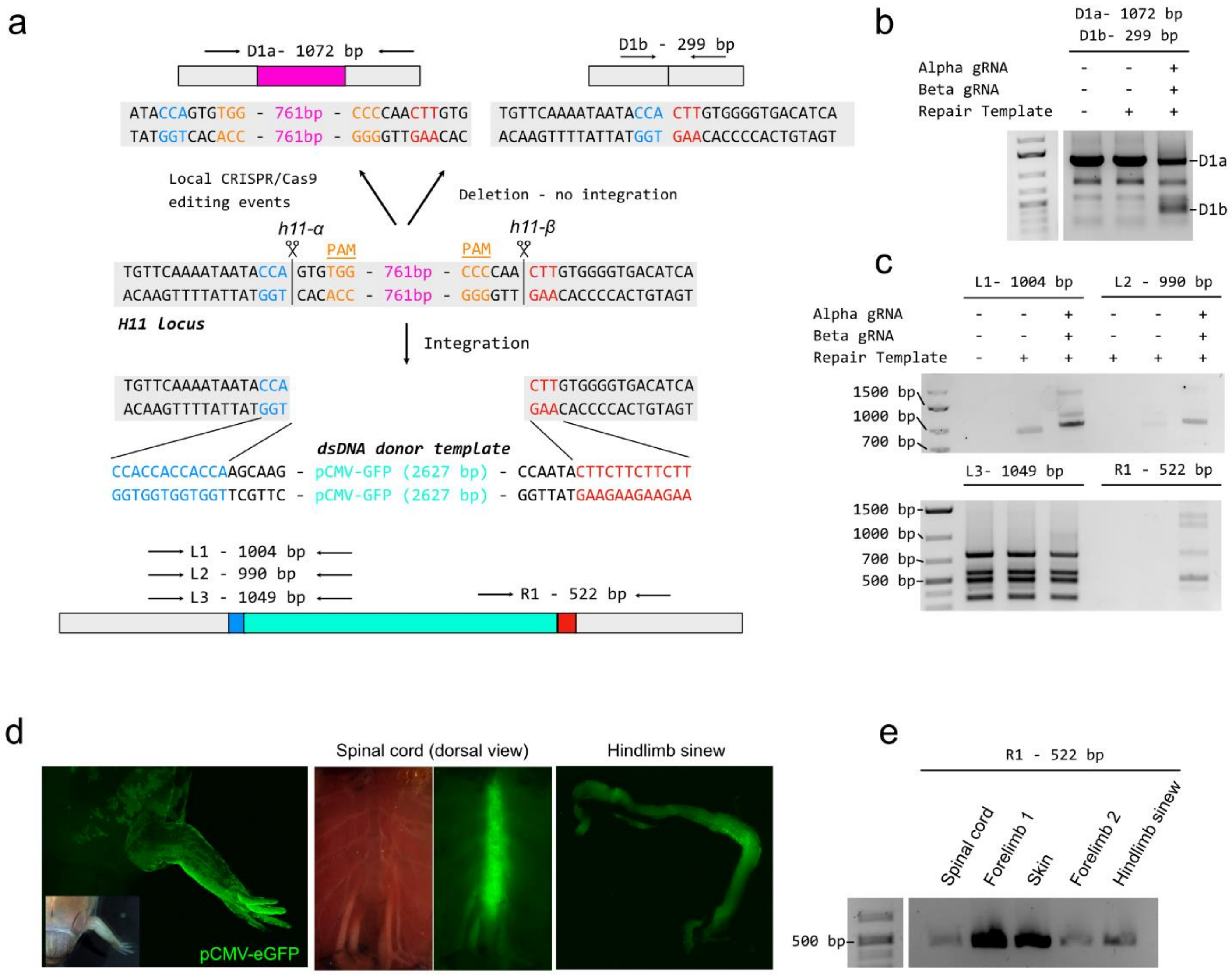

Supplementary figure 8: Stable integration into the *X. tropicalis hipp11* locus. (a) Schematic of the CRISPR/Cas9 integration strategy. Co-delivering two RNPs (*h11-α* and *h11-β*) together with dsDNA donor template leads to several potential gene editing outcomes. Firstly D1a, amplified are unedited and locally edited (Indels) genome copies. Secondly D1b, amplified in case of CRISPR-mediated deletion of intervening DNA between the binding site of *h11-α* and *h11-β*. Thirdly, integration of the dsDNA donor template, in place of the intervening DNA between the binding site of *h11-α* and *h11-β*. **(b)** Co-delivering *h11-α* and *h11-β* RNP results in a lower amplification of product D1a, and an increase in product D1b, showcasing CRISPR-mediated deletion as an editing outcome. All lanes are pools of 50 injected embryos. **(c)** 5' (L1, L2, L3) and 3' (R1) junction products can be amplified, revealing targeted integration of pCMV-eGFP in the *hipp11* locus. **(d)** Example of clonal expansion of GFP+ cells as an embryo develops from neurula to metamorphosis. **(e)** Several GFP+ tissues were dissected, lysed and the 3' (R1) junction products amplified, revealing stable long-term integration into the *hipp11* locus

bioRxiv preprint doi: https://doi.org/10.1101/2024.09.23.614424; this version posted September 23, 2024. The copyright holder for this preprint (which was not certified by peer review) is the author/funder, who has granted bioRxiv a license to display the preprint in perpetuity. It is made available under aCC-BY 4.0 International license.

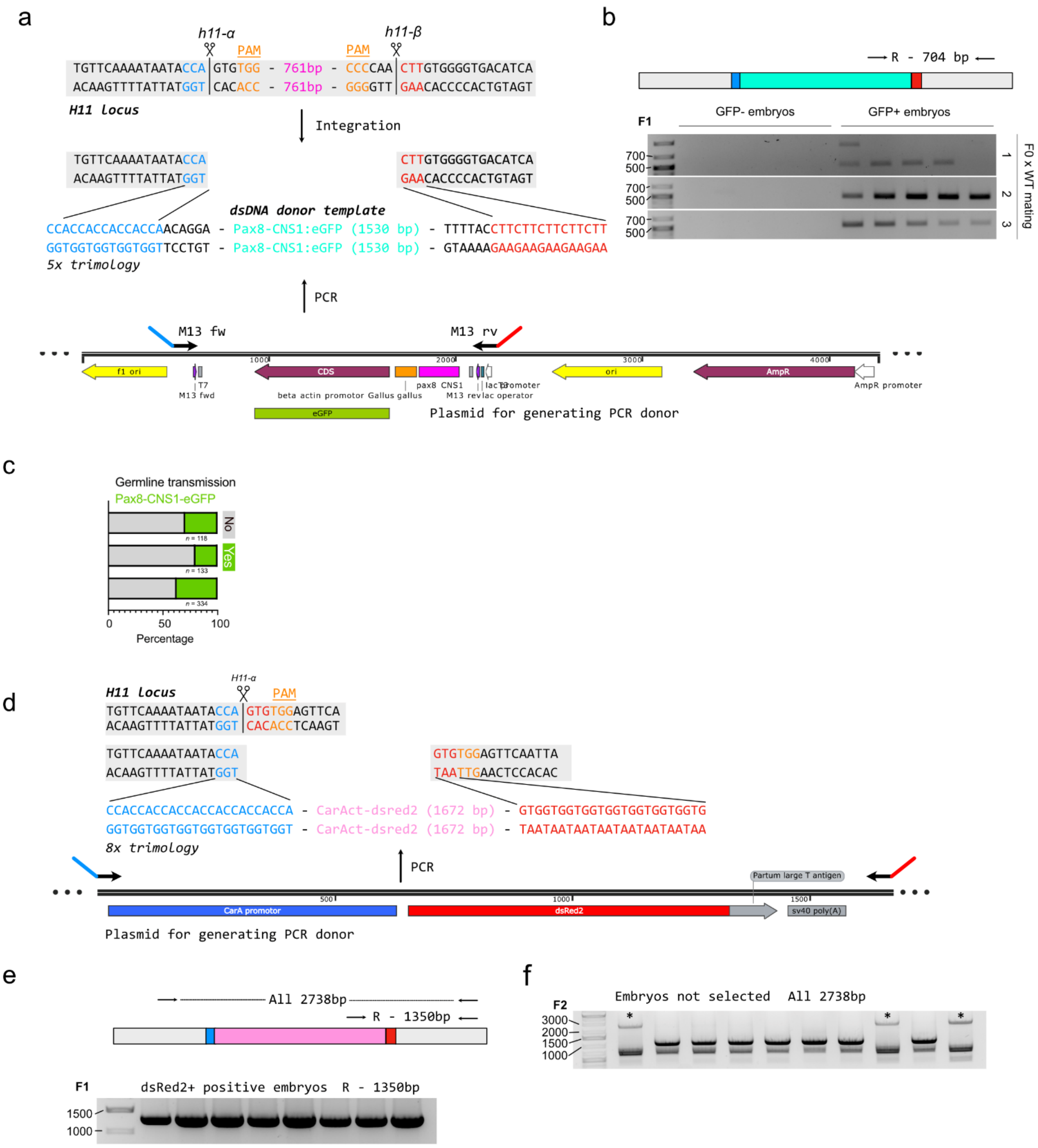

Supplementary figure 9: Stable integration into and tissue-specific expression from the *X. tropicalis hipp11* locus. (a) Schematic CRISPR/Cas9 integration strategy. Co-delivering two RNPs (*h11-α* and *h11-β*) together with Pax8-CNS1:eGFP dsDNA donor template (5x trimology) generated by simple overhang PCRs from a plasmid **(b)** In F1 offspring, 3' (R) junction products were amplified, revealing targeted integration of Pax8-CNS1:eGFP in the *hipp11* locus and positive germline transmission. **(c)** In six F0 founder animals, three (50%) demonstrated transmission through germline at mean rate of 29.1% ± 8.6%. **(d)** Schematic CRISPR/Cas9 integration strategy. Delivering two RNPs (*h11-α*) together with CarAct:dsRed2 dsDNA donor template (8x trimology) generated by simple overhang PCRs from a plasmid. **(e)** In F1 offspring, 3' (R) junction products were amplified, revealing targeted integration of Pax8-CNS1:eGFP in the *hipp11* locus and positive germline transmission. **(f)** F2 homozygote knock-in animals (stars) can be identified by a PCR with primers binding the genomic sites left and right of *h11-α*, thus amplifying the entire fragment (2738 bp) in homozygotes knock-in animals.

bioRxiv preprint doi: https://doi.org/10.1101/2024.09.23.614424; this version posted September 23, 2024. The copyright holder for this preprint (which was not certified by peer review) is the author/funder, who has granted bioRxiv a license to display the preprint in perpetuity. It is made available under aCC-BY 4.0 International license.

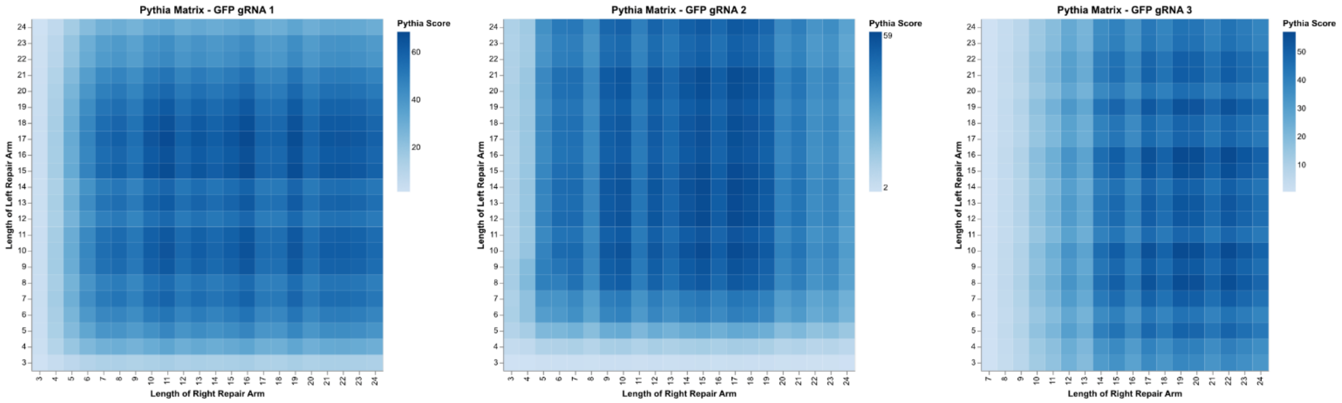

Supplementary Figure 10: Pythia Matrices for GFP gRNAs.

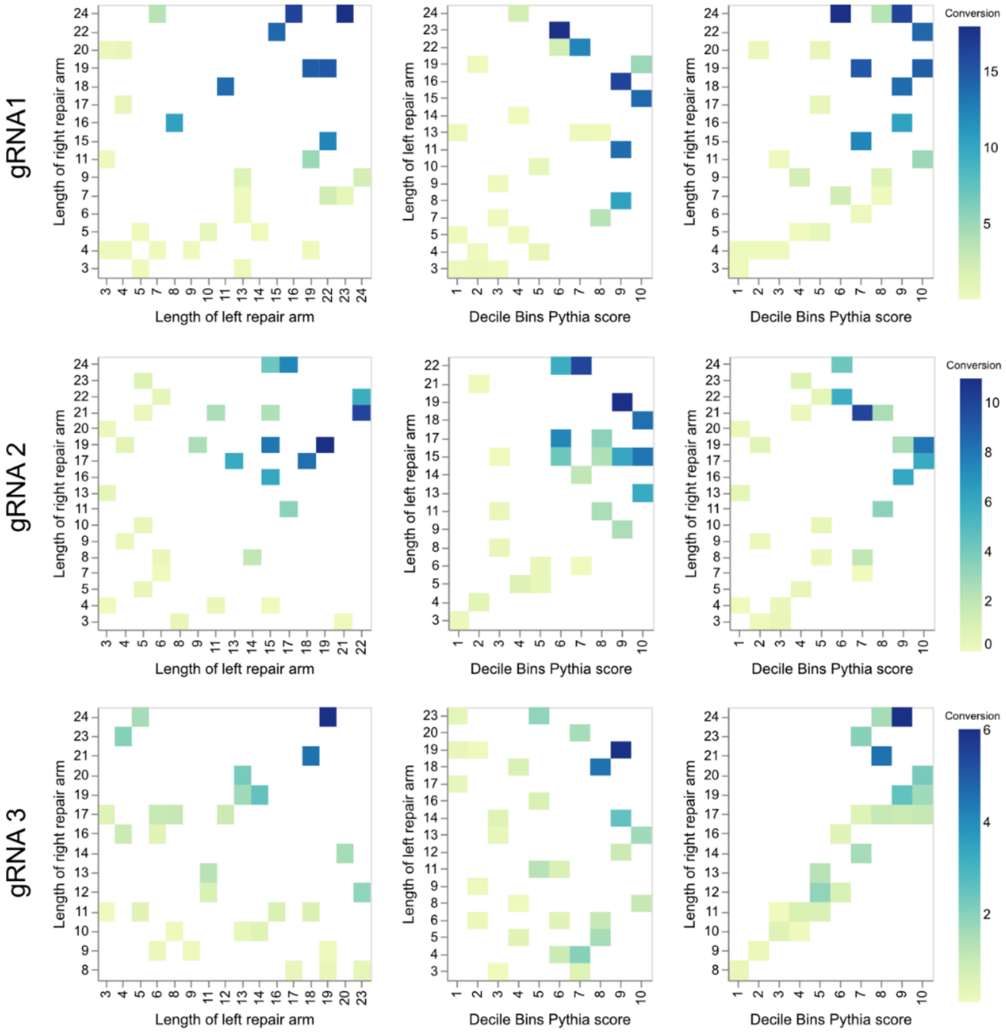

Supplementary Figure 11: Relationship between lengths of repair arms, Pythia scores and eGFP- to eBFP conversion efficiencies. Each row represents one GFP gRNA (top 1, middle 2, bottom 3) each with 30 distinct repair templates binned across Pythia prediction score (predicted %perfect repair). Both left and right repair arm length is related to conversion (eGFP- to eBFP) efficiencies. Further, there is a direct correlation between Pythia prediction scores and conversion efficiencies.

bioRxiv preprint doi: https://doi.org/10.1101/2024.09.23.614424; this version posted September 23, 2024. The copyright holder for this preprint (which was not certified by peer review) is the author/funder, who has granted bioRxiv a license to display the preprint in perpetuity. It is made available under aCC-BY 4.0 International license.

Supplementary Movies

Supplementary Movie 1: Benchtop mesoSPIM recording of an adult kidney from an F0 *Xenopus tropicalis* with a stable integration of pax8-CNS1:eGFP in the *hipp11* stable landing site.

Supplementary Movie 2: Time-lapse imaging of tadpole development in a stable F1 *Xenopus tropicali*s line with a stable integration of pax8-CNS1:eGFP in the *hipp11* stable landing site.

Supplementary Movie 3: Time-lapse imaging of tadpole development in a stable F1 *Xenopus tropicali*s with a stable integration of CarAct:dsRed2 in the *hipp11* stable landing site.

Supplementary Movie 4: Benchtop mesoSPIM and Schmidt objective two-photon microscopy recording of a stable F2 *Xenopus tropicali*s with a stable integration of CarAct:dsRed2 in the *hipp11* stable landing site.

Supplementary Movie 5: Benchtop mesoSPIM recording of wildDisco processed adult mouse brain demonstrating viral delivery and in-frame eGFP-tagging of *Tubb2a*.

bioRxiv preprint doi: https://doi.org/10.1101/2024.09.23.614424; this version posted September 23, 2024. The copyright holder for this preprint (which was not certified by peer review) is the author/funder, who has granted bioRxiv a license to display the preprint in perpetuity. It is made available under aCC-BY 4.0 International license.

Supplementary Tables

Supplementary Table 1: Primer sequences used for PCR amplification

Figure	Subpanel	Forward primer	Reverse primer
Fig. 3c	*Top*	5'-ATG CCC CAA TCT ATG CCC TG-3'	5'-GAT CCG CCA CAA CAT CGA GG-3'
	Bottom	5'-ACA TCT GTG TCG CAT GGA GT-3'	5'-CGT AGA TGT ACT GCC AAG TAG G-3'
Fig. 3f	*Top*	5'-GAT CCG CCA CAA CAT CGA GG-3'	5'-ATG CCC CAA TCT ATG CCC TG-3'
	Bottom	5'-ACA TCT GTG TCG CAT GGA GT-3'	5'-CGT AGA TGT ACT GCC AAG TAG G-3'
Fig. 4f	na	5'-ACA CTC TTT CCC TAC ACG ACG CTC TTC CGA TCT CCT CAA GAT GTC AGC CAC CT-3'	5'-GAC TGG AGT TCA GAC GTG TGC TCT TCC GAT CTG CAG ATG AAC TTC AGG GTC AG-3'
Fig. 5e	na	5'-ACA TCT AAG GCC CAT CCA TTA CC-3'	5'-ATA ACT CGC TGC ATG TGT TCC T-3'
Fig. S2b	Left	5'-CGT AGA TGT ACT GCC AAG TAG G-3'	5'-CTG TGC TAG CTC TTC CAG CC-3'
	Right	5'-CTA TAT CAT GGC CGA CAA GCA-3'	5'-CTC ACA AAG GGA GTT TTC CAC A-3'
Fig. S2c	Left	5'-CGT AGA TGT ACT GCC AAG TAG G-3'	5'-CTG TGC TAG CTC TTC CAG CC-3'
	Right	5'-CTA TAT CAT GGC CGA CAA GCA-3'	5'-CTC ACA AAG GGA GTT TTC CAC A-3'
Fig. S2d	Left	5'-CGT AGA TGT ACT GCC AAG TAG G-3'	5'-CTG TGC TAG CTC TTC CAG CC-3'
	Right	5'-CTA TAT CAT GGC CGA CAA GCA-3'	5'-CTC ACA AAG GGA GTT TTC CAC A-3'
Fig. S3a	Left	5'-CGT AGA TGT ACT GCC AAG TAG G-3'	5'-CTG TGC TAG CTC TTC CAG CC-3'
	Right	5'-CTA TAT CAT GGC CGA CAA GCA-3'	5'-CTC ACA AAG GGA GTT TTC CAC A-3'
	NGS - Left	5'-ACA CTC TTT CCC TAC ACG ACG CTC TTC CGA TCT GCC ACT AGG GAC AGG ATT GG-3'	5'-GAC TGG AGT TCA GAC GTG TGC TCT TCC GAT CTT AGG GGG CGT ACT TGG CAT A-3'
	NGS - Right	5'-ACA CTC TTT CCC TAC ACG ACG CTC TTC CGA TCT GGA GGT TAA AGT TTT GCT ATG CTG T-3'	5'-GAC TGG AGT TCA GAC GTG TGC TCT TCC GAT CTT GCT TCT CCT CTT GGG AAG TG-3'
Fig. S8b	na	5'-ACA TCT GTG TCG CAT GGA GT-3'	5'-ATG CCC CAA TCT ATG CCC TG-3'
Fig. S8c	Left 1	5'-GAT CCG CCA CAA CAT CGA GG-3'	5'-ATG CCC CAA TCT ATG CCC TG-3'
	Left 2	5'-GAT CCG CCA CAA CAT CGA GG-3'	5'-CGC TGC CCT TAA GGA GCT AAA-3'
	Left 3	5'-ACA AGC AGA AGA ACG GCA TC-3'	5'-ATG CCC CAA TCT ATG CCC TG-3'
	Right 1	5'-ACA TCT GTG TCG CAT GGA GT-3'	5'-CGT AGA TGT ACT GCC AAG TAG G-3'
Fig. S8e	na	5'-ATT CAG TGT CTT TGT TGA ATC ATT G-3'	5'-GGA AAG TCC CAT AAG GTC ATG-3'
Fig. S9b	na	5'-GAT CCG CCA CAA CAT CGA GG-3'	5'-ACA TCT GTG TCG CAT GGA GT-3'
Fig. S9e	na	5'-ATG CCC CAA TCT ATG CCC TG-3'	5'-ACA TCT GTG TCG CAT GGA GT-3'
Fig. S9f	na	5'-ATG CCC CAA TCT ATG CCC TG-3'	5'-ACA TCT GTG TCG CAT GGA GT-3'

bioRxiv preprint doi: https://doi.org/10.1101/2024.09.23.614424; this version posted September 23, 2024. The copyright holder for this preprint (which was not certified by peer review) is the author/funder, who has granted bioRxiv a license to display the preprint in perpetuity. It is made available under aCC-BY 4.0 International license.

Supplementary Table 2: Sequences, CRISPRScan scores and InDelphi metrics for gRNAs

Species	Target name	Target sequence	Identifier (S = C/G, W=A/T)	CRISPR_Scan_Score	MH_del_frequency
Human	*AAVS1*	TCTAACCCCCACCTCCTGTT	na	55	not calculated
Human	EMILIN2	TTGTAAGGAATGCACGCAGG	SSS_AGG	81	49.53216415
Human	OBSL1	AGGGTTCGGAGCACCCCAGG	SSS_AGG	86	57.19376868
Human	PPP1R14A	TCGCGGGCCCGCGGGCCAGG	SSS_AGG	93	70.51902761
Human	NTN5	GCAGGTGCTAGCGTCCGAGG	SSS_AGG	91	82.41066334
Human	ADARB2	AGGCCGGTTGTGCCGGTAGG	SSW_AGG	94	49.72957086
Human	ONECUT3	GTGGCCTGGCGGGCCGTAGG	SSW_AGG	88	57.20800631
Human	KCNS1	GCCTCAGGCTGCATCCTAGG	SSW_AGG	82	74.68993556
Human	IL17RA	CCGCTGGTCGGGGAGGAAGG	SSW_AGG	85	84.79010482
Human	CKMT1A	GTGGACACTGCTGCCACAGG	SWS_AGG	90	48.41241661
Human	PHACTR2	GAAGGTGCTGGAGCCACAGG	SWS_AGG	88	57.03681447
Human	TBC1D25	GGCGGCGGCGGCCGCTGAGG	SWS_AGG	81	72.23344544
Human	SMIM24	TGGCCTTGGAACACCAGAGG	SWS_AGG	91	81.93408194
Human	CD164L2	CAGCCAGCTGGGCACATAGG	SWW_AGG	83	46.17176194
Human	AGBL4	AGGAGTGGGTGCTTGTAAGG	SWW_AGG	81	59.7767934
Human	AMER3	GCGCAGCTGGACCCCAAAGG	SWW_AGG	82	74.21624543
Human	ESX1	GGTGGCCTGGGTGGCATAGG	SWW_AGG	81	80.21881908
Human	ANO2	AGGAGTACTGGTACACCAGG	WSS_AGG	84	47.12660918
Human	P3H4	TGAAGGGGCCCATGAGCAGG	WSS_AGG	81	59.34548122
Human	RBP3	TCTGGAGAGGGGACAGCAGG	WSS_AGG	87	70.19363343
Human	LRRK1	AGGGAGGGACTGTGTCGAGG	WSS_AGG	84	83.23986201
Human	ASB18	CGGCGCCGTGCCGCAGTAGG	WSW_AGG	90	49.78313429
Human	ABHD14B	CCGGGTGCTGATCATGAAGG	WSW_AGG	92	57.31921541
Human	ANO3	AGGCTTGATGTTGGAGAAGG	WSW_AGG	92	74.51597946
Human	SIAH3	CCGCCTCCTGGTGGTGAAGG	WSW_AGG	84	84.99531349
Human	ANKS6	CAGCCCCGTGATCCAACAGG	WWS_AGG	88	46.64236335
Human	CPT1C	TCAGGCGGTGGCGGTACAGG	WWS_AGG	83	56.34941667
Human	NACAD	CAGCAGGCTGAGGGAACAGG	WWS_AGG	81	70.2468907
Human	PLXNB3	CGGGGCTGAGCTGGAAGAGG	WWS_AGG	88	83.62428374
Human	ZNF174	TCAGTGGGTGGCTTTTTAGG	WWW_AGG	82	47.12038193
Human	WDFY4	CCCCCTGGGAGCCCAAAAGG	WWW_AGG	81	59.77036617
Human	NLRP4	TCTGGAGGAGCTCAAAAAGG	WWW_AGG	82	74.50260314
Human	TMEM132C	CGGGGGGATGGTGGTAAAGG	WWW_AGG	85	85.80079197
Xenopus	*h11*- alpha	TGTTCAAAATAATACCAGTG	na	58	not calculated
Xenopus	*h11*- beta	ACTACAGTGGGGTGTTCAAC	na	49	not calculated
Mus Musculus	*Tubb2a*	GGGCGAGTTCGAGGAGGAGG	na	76	not calculated
Specie-Agnostic	GFP1	CTCGTGACCACCCTGACCTA	na	63	not calculated
Specie-Agnostic	GFP2	AGCACTGCACGCCGTAGGTC	na	40	not calculated
Specie-Agnostic	GFP3	GCTGAAGCACTGCACGCCGT	na	54	not calculated

bioRxiv preprint doi: https://doi.org/10.1101/2024.09.23.614424; this version posted September 23, 2024. The copyright holder for this preprint (which was not certified by peer review) is the author/funder, who has granted bioRxiv a license to display the preprint in perpetuity. It is made available under aCC-BY 4.0 International license.

Supplementary Table 3: Primer sequences used for amplification of pCMV-eGFP repair cassette from AAV-CMV-GFP (addgene #67634) for each of the 32 target loci.

**Oligo Name**	**DNA Sequence (5'-3')**
EMILIN2_Screen_fw	CGC CGC CGC CGC CGC TAT TGG CTC ATG TCC AAC
OBSL1_Screen_fw	CCC CCC CCC CCC CCC TAT TGG CTC ATG TCC AAC
PPP1R14A_Screen_fw	GCC GCC GCC GCC GCC TAT TGG CTC ATG TCC AAC
NTN5_Screen_fw	CCG CCG CCG CCG CCG TAT TGG CTC ATG TCC AAC
IL17RA_Screen_fw	GGA GGA GGA GGA GGA TAT TGG CTC ATG TCC AAC
KCNS1_Screen_fw	CCT CCT CCT CCT CCT TAT TGG CTC ATG TCC AAC
ONECUT3_Screen_fw	CGT CGT CGT CGT CGT TAT TGG CTC ATG TCC AAC
ADARB2_Screen_fw	GGT GGT GGT GGT GGT TAT TGG CTC ATG TCC AAC
CKMT1A_Screen_fw	CAC CAC CAC CAC CAC TAT TGG CTC ATG TCC AAC
PHACTR2_Screen_fw	CAC CAC CAC CAC CAC TAT TGG CTC ATG TCC AAC
TBC1D25_Screen_fw	CTG CTG CTG CTG CTG TAT TGG CTC ATG TCC AAC
CD164L2_Screen_fw	CAT CAT CAT CAT CAT TAT TGG CTC ATG TCC AAC
AGBL4_Screen_fw	GTA GTA GTA GTA GTA TAT TGG CTC ATG TCC AAC
AMER3_Screen_fw	CAA CAA CAA CAA CAA TAT TGG CTC ATG TCC AAC
ESX1_Screen_fw	CAT CAT CAT CAT CAT TAT TGG CTC ATG TCC AAC
ANO2_Screen_fw	ACC ACC ACC ACC ACC TAT TGG CTC ATG TCC AAC
P3H4_Screen_fw	AGC AGC AGC AGC AGC TAT TGG CTC ATG TCC AAC
RBP3_Screen_fw	AGC AGC AGC AGC AGC TAT TGG CTC ATG TCC AAC
LRRK1_Screen_fw	TCG TCG TCG TCG TCG TAT TGG CTC ATG TCC AAC
ANO3_Screen_fw	AGA AGA AGA AGA AGA TAT TGG CTC ATG TCC AAC
ABHD14B_Screen_fw	TGA TGA TGA TGA TGA TAT TGG CTC ATG TCC AAC
ASB18_Screen_fw	AGT AGT AGT AGT AGT TAT TGG CTC ATG TCC AAC
ANKS6_Screen_fw	AAC AAC AAC AAC AAC TAT TGG CTC ATG TCC AAC
CPT1C_Screen_fw	TAC TAC TAC TAC TAC TAT TGG CTC ATG TCC AAC
PLXNB3_Screen_fw	AAG AAG AAG AAG AAG TAT TGG CTC ATG TCC AAC
NACAD_Screen_fw	AAC AAC AAC AAC AAC TAT TGG CTC ATG TCC AAC
ZNF174_Screen_fw	TTT TTT TTT TTT TTT TAT TGG CTC ATG TCC AAC
NLRP4_Screen_fw	AAA AAA AAA AAA AAA TAT TGG CTC ATG TCC AAC
TMEM132C_Screen_fw	TAA TAA TAA TAA TAA TAT TGG CTC ATG TCC AAC
SMIM24_Screen_fw	CAG CAG CAG CAG CAG TAT TGG CTC ATG TCC AAC
SIAH3_Screen_fw	TGA TGA TGA TGA TGA TAT TGG CTC ATG TCC AAC
WDFY4_Screen_fw	AAA AAA AAA AAA AAA TAT TGG CTC ATG TCC AAC
Screen_rv	CCT CCT CCT CCT CCT AGC AAG AGA ACT GAG TGG

bioRxiv preprint doi: https://doi.org/10.1101/2024.09.23.614424; this version posted September 23, 2024. The copyright holder for this preprint (which was not certified by peer review) is the author/funder, who has granted bioRxiv a license to display the preprint in perpetuity. It is made available under aCC-BY 4.0 International license.

Supplementary Table 4: Sequences of ssODN repair templates used for eGFP -> eBFP conversion in HEK293T cells.

Oligo Name	DNA Sequence (5'-3')	Modifications	Scale
GFP1_0-10_1	GAG CCA C	none	0.2 µmol
GFP1_0-10_2	TGA CCA CCC TGA GCC A	none	Genomics
GFP1_0-10_3	CTG AGC CA	none	0.2 µmol
GFP1_10-20_1	TGA GCC AC	none	0.2 µmol
GFP1_10-20_2	CCC TCG TGA CCA CCC TGA GCC AC	none	Genomics
GFP1_10-20_3	GAG CCA CGG CGT GCA GTG CTT CA	none	Genomics
GFP1_20-30_1	GAG CCA CGG CGT GC	none	Genomics
GFP1_20-30_2	CCC TGA GCC AC	none	0.2 µmol
GFP1_20-30_3	CAC CCT GAG CCA C	none	Genomics
GFP1_30-40_1	GCC CAC CCT CGT GAC CAC CCT GAG CCA CGG CGT	none	Genomics
GFP1_30-40_2	CTG AGC CAC G	none	0.2 µmol
GFP1_30-40_3	GTG ACC ACC CTG AGC CAC G	none	Genomics
GFP1_40-50_1	CCA CCC TGA GCC ACG	none	Genomics
GFP1_40-50_2	TGA GCC ACG GCG TGC AGT GCT	none	Genomics
GFP1_40-50_3	TGA GCC ACG GCG TGC AGT GCT TCA	none	Genomics
GFP1_50-60_1	CCC ACC CTC GTG ACC ACC CTG AGC CAC GGC	none	Genomics
GFP1_50-60_2	CCC ACC CTC GTG ACC ACC CTG AGC CAC GGC GTG CAG TGC TTC AGC CG	none	Genomics
GFP1_50-60_3	CCA CCC TCG TGA CCA CCC TGA GCC ACG GC	none	Genomics
GFP1_60-70_1	TGA CCA CCC TGA GCC ACG G	none	Genomics
GFP1_60-70_2	CCA CCC TCG TGA CCA CCC TGA GCC ACG GCG TGC AGT GCT TC	none	Genomics
GFP1_60-70_3	CCA CCC TCG TGA CCA CCC TGA GCC ACG GCG TGC AGT G	none	Genomics
GFP1_70-80_1	TGA CCA CCC TGA GCC ACG GCG T	none	Genomics
GFP1_70-80_2	CCC TGA GCC ACG GCG TGC AGT GCT TCA GCC G	none	Genomics
GFP1_70-80_3	TGA CCA CCC TGA GCC ACG GC	none	Genomics
GFP1_80-90_1	ACC ACC CTG AGC CAC GGC GTG CAG TGC TT	none	Genomics
GFP1_80-90_2	TCG TGA CCA CCC TGA GCC ACG GCG TGC AGT GCT TCA GCC G	none	Genomics
GFP1_80-90_3	ACC CTG AGC CAC GGC GTG CAG TGC	none	Genomics
GFP1_90-100_1	CGT GAC CAC CCT GAG CCA CGG CGT GCA GTG CTT CAG C	none	Genomics
GFP1_90-100_2	CCC TCG TGA CCA CCC TGA GCC ACG GCG TGC AGT GCT TC	none	Genomics
GFP1_90-100_3	CCC TCG TGA CCA CCC TGA GCC ACG GCG TGC	none	Genomics
GFP2_0-10_1	TGG CTC AGG GTG GTC A	none	Genomics
GFP2_0-10_2	TGG CTC A	none	0.2 µmol
GFP2_0-10_3	TGG CTC AGG GTG GTC ACG AGG GT	none	Genomics
GFP2_10-20_1	GTG GCT CAG GGT G	none	Genomics
GFP2_10-20_2	GTG GCT CAG GGT GGT CAC GAG GG	none	Genomics
GFP2_10-20_3	CTG AAG CAC TGC ACG CCG TGG CTC	none	Genomics
GFP2_20-30_1	CAC TGC ACG CCG TGG CTC A	none	Genomics
GFP2_20-30_2	CGC CGT GGC TC	none	0.2 µmol
GFP2_20-30_3	GCA CGC CGT GGC TCA	none	Genomics
GFP2_30-40_1	CGT GGC TCA G	none	0.2 µmol
GFP2_30-40_2	CGT GGC TCA GGG TGG TCA CGA GGG TG	none	Genomics
GFP2_30-40_3	CGT GGC TCA GGG TGG TCA CGA GGG TGG G	none	Genomics
GFP2_40-50_1	CCG TGG CTC AGG GTG GTC ACG AGG GTG G	none	Genomics
GFP2_40-50_2	CCG TGG CTC AGG GT	none	Genomics
GFP2_40-50_3	CGT GGC TCA GGG TGG	none	Genomics
GFP2_50-60_1	GCT GAA GCA CTG CAC GCC GTG GCT CAG GGT GGT CAC GAG GGT GG	none	Genomics
GFP2_50-60_2	AGC ACT GCA CGC CGT GGC TCA GGG TGG TCA CGA GGG TGG GC	none	Genomics
GFP2_50-60_3	CAC TGC ACG CCG TGG CTC AGG GTG GTC ACG AGG GTG GGC	none	Genomics
GFP2_60-70_1	GCT GAA GCA CTG CAC GCC GTG GCT CAG GGT GGT CAC GAG GGT G	none	Genomics
GFP2_60-70_2	CCG TGG CTC AGG G	none	Genomics
GFP2_60-70_3	ACT GCA CGC CGT GGC TCA GGG T	none	Genomics

bioRxiv preprint doi: https://doi.org/10.1101/2024.09.23.614424; this version posted September 23, 2024. The copyright holder for this preprint (which was not certified by peer review) is the author/funder, who has granted bioRxiv a license to display the preprint in perpetuity. It is made available under aCC-BY 4.0 International license.

GFP2_70-80_1	CAC TGC ACG CCG TGG CTC AGG GTG GTC ACG AGG GTG	none	Genomics
GFP2_70-80_2	AGC ACT GCA CGC CGT GGC TCA GGG TGG T	none	Genomics
GFP2_70-80_3	GCA CGC CGT GGC TCA GGG TGG TCA CGA GGG TG	none	Genomics
GFP2_80-90_1	GAA GCA CTG CAC GCC GTG GCT CAG GGT GGT CAC GAG GG	none	Genomics
GFP2_80-90_2	CAC TGC ACG CCG TGG CTC AGG GTG GTC ACG A	none	Genomics
GFP2_80-90_3	ACG CCG TGG CTC AGG GTG GTC ACG AGG G	none	Genomics
GFP2_90-100_1	AAG CAC TGC ACG CCG TGG CTC AGG GTG GTC ACG AG	none	Genomics
GFP2_90-100_2	CTG CAC GCC GTG GCT CAG GGT GGT CAC GAG	none	Genomics
GFP2_90-100_3	CAC TGC ACG CCG TGG CTC AGG GTG GTC ACG AGG G	none	Genomics
GFP3_0-10_1	GCT GAA GCA CTG CAC GCC GTG GCT C	none	Genomics
GFP3_0-10_2	GTA GCG GCT GAA GCA CTG CAC GCC GTG GCT C	none	Genomics
GFP3_0-10_3	CGG CTG AAG CAC TGC ACG CCG TGG CTC	none	Genomics
GFP3_10-20_1	CGG CTG AAG CAC TGC ACG CCG TGG CTC A	none	Genomics
GFP3_10-20_2	ACT GCA CGC CGT GGC TCA	none	Genomics
GFP3_10-20_3	GCA CGC CGT GGC TCA	none	Genomics
GFP3_20-30_1	CGC CGT GGC TCA GG	none	Genomics
GFP3_20-30_2	AAG CAC TGC ACG CCG TGG CTC AG	none	Genomics
GFP3_20-30_3	GAA GCA CTG CAC GCC GTG GCT CAG	none	Genomics
GFP3_30-40_1	CAC GCC GTG GCT CAG G	none	Genomics
GFP3_30-40_2	GGC TGA AGC ACT GCA CGC CGT GGC TCA GG	none	Genomics
GFP3_30-40_3	CTG CAC GCC GTG GCT CAG	none	Genomics
GFP3_40-50_1	GTA GCG GCT GAA GCA CTG CAC GCC GTG GCT CAG GG	none	Genomics
GFP3_40-50_2	GCA CTG CAC GCC GTG GCT CAG GGT	none	Genomics
GFP3_40-50_3	CTG AAG CAC TGC ACG CCG TGG CTC AGG	none	Genomics
GFP3_50-60_1	ACG CCG TGG CTC AGG GTG GT	none	Genomics
GFP3_50-60_2	GCA CGC CGT GGC TCA GGG TGG T	none	Genomics
GFP3_50-60_3	GCA CTG CAC GCC GTG GCT CAG GG	none	Genomics
GFP3_60-70_1	CGC CGT GGC TCA GGG TGG TC	none	Genomics
GFP3_60-70_2	GCG GCT GAA GCA CTG CAC GCC GTG GCT CAG GGT G	none	Genomics
GFP3_60-70_3	ACG CCG TGG CTC AGG GTG GTC ACG AGG	none	Genomics
GFP3_70-80_1	GGC TGA AGC ACT GCA CGC CGT GGC TCA GGG TGG TCA CGA	none	Genomics
GFP3_70-80_2	CAC GCC GTG GCT CAG GGT GGT CAC GAG GG	none	Genomics
GFP3_70-80_3	GCA CGC CGT GGC TCA GGG TGG TC	none	Genomics
GFP3_80-90_1	AGC ACT GCA CGC CGT GGC TCA GGG TGG TC	none	Genomics
GFP3_80-90_2	CGG CTG AAG CAC TGC ACG CCG TGG CTC AGG GTG GTC ACG AGG G	none	Genomics
GFP3_80-90_3	GAA GCA CTG CAC GCC GTG GCT CAG GGT GGT CAC	none	Genomics
GFP3_90-100_1	AAG CAC TGC ACG CCG TGG CTC AGG GTG GTC ACG	none	Genomics
GFP3_90-100_2	CTG CAC GCC GTG GCT CAG GGT GGT C	none	Genomics
GFP3_90-100_3	AAG CAC TGC ACG CCG TGG CTC AGG GTG GTC AC	none	Genomics
